# The Mechanisms of Cardiac Protection Using a Synthetic Agonist of Galanin Receptors during Chronic Administration of Doxorubicin

**DOI:** 10.32607/actanaturae.10945

**Published:** 2020

**Authors:** I. M. Studneva, O. M. Veselova, A. A. Bahtin, G. G. Konovalova, V. Z. Lankin, O. I. Pisarenko

**Affiliations:** National Medical Research Center for Cardiology, Moscow, 121552 Russia; Research and Clinical Center of Otorhinolaryngology, Moscow, 123182 Russia

**Keywords:** galanin, rat, doxorubicin, myocardial metabolism, antioxidant enzymes

## Abstract

The use of the anticancer drug doxorubicin (Dox) is limited by its cardiotoxic effect. The aim of this work was to study the effect of a new synthetic agonist of the galanin receptor GalR1-3 [βAla14, His15]-galanine (2–15) (G) on the metabolism, antioxidant enzyme activity, and cardiac function in rats with cardiomyopathy (CM) caused by chronic administration of Dox. Coadministration of peptide G and Dox significantly increased the fractional shortening (FS) and ejection fraction (EF) by an average of 30 ± 4% compared with the indices in the Dox group. The reduced severity of cardiac dysfunction under the action of G was accompanied by a 2.5-fold decrease in the activity of creatine kinase-MB (CK-MB) in blood plasma. The protective mechanism of the action of peptide G is caused by a reduced lipid peroxidation (LP) that is due to the increased activity of Cu,Zn superoxide dismutase (Cu,Zn-SOD) and glutathione peroxidase (GSH-Px) in the damaged heart. Administration of peptide G significantly increased the adenine nucleotide pool (ΣAH), ATP content, and the levels of phosphocreatine (PCr) and total creatine (ΣCr) in the damaged myocardium. It also reduced lactate accumulation relative to its content in the Dox group. The better energy supply of cardiomyocytes after treatment with peptide G prevented the accumulation of cytotoxic ammonia and disruption in the metabolism of the key myocardial amino acids (glutamic acid (Glu), aspartic acid (Asp), and alanine (Ala)). Peptide G significantly improved the morphological parameters of the heart in rats treated with Dox. The results show promise in using peptide G to efficiently correct functional, morphological, and metabolic damage to the heart caused by anthracycline chemotherapy.

## INTRODUCTION

Reduced energy production in cardiomyocytes that is due to mitochondrial dysfunction or the toxic effects of drugs can cause chronic damage to the myocardium. Doxorubicin (Dox), an anthracycline anticancer drug, can induce cardiomyopathy (CM) and congestive heart failure. So, its application in oncologic practice is limited [1]. Dox-induced cardiotoxicity is a multifactorial process that may bring about the death of cardiomyocytes and endothelial cells [2]. The key factors that accompany Dox-induced cardiotoxicity include impaired oxidative phosphorylation and the generation of reactive oxygen species (ROS), which initiate lipid peroxidation (LP) [3]. Neither optimization of the administration regimes of Dox nor the use of liposomal forms of this drug can eliminate its high cardiotoxicity [4]. In this context, developing approaches that could help prevent or mitigate Dox-induced cardiac damage appears relevant. We previously showed that exogenous N-terminal fragments of galanin (2–11) and (2–15) bind to the GalR2 receptor and protect cardiomyocytes from ischemia/reperfusion (I/R) injury [5, 6]. The protective effects of these peptides are associated with a decreased formation of mitochondrial superoxide radicals and the triggering of signaling cascades, which reduce apoptotic and necrotic cell death [5, 6]. Subsequently, we synthesized a number of peptide analogs of galanin fragments (2–11) and (2–15), where the pharmacophore amino acid residues responsible for the binding to the GalR2 receptor were preserved. These peptides were tested using models of myocardial I/R injury and were found to exhibit cardioprotective activity [7]. A chimeric molecule that has a sequence of galanin (2–13) supplemented by natural dipeptide carnosine, H-Trp-Thr-Leu-Asn-Ser-Ala-Gly-Tyr- Leu-Leu-Gly-Pro-βAla-His-OH (G), turned out to be the most effective one [8]. Peptide G improved cardiac function, membrane integrity, and the energy state of cardiomyocytes in rats after long-term administration of Dox [9], thus directly indicating that it affects myocardial metabolism. However, the mechanisms of action of this compound remain poorly understood. The objective of this study was to evaluate the effect of peptide G on less studied Dox targets (activity of antioxidant enzymes and indices of nitrogenous and carbohydrate metabolism) in the heart of rats with Dox-induced cardiomyopathy. The cardiotoxic effect of Dox was characterized by changes in the creatine kinase-MB (CK-MB) activity in blood, the level of oxidative stress in the heart and blood plasma of animals, as well as the morphological state of the myocardium.

## MATERIALS AND METHODS

**Experimental design**

The experiments were performed using male Wistar rats weighing 280–300 g obtained from the Stolbovaya nursery (Moscow region, Russia). To simulate cardiomyopathy, Dox was administered according to a regiment similar to that described in [9]. The animals were divided into four groups. The control group (C) received an intraperitoneal injection of saline (1 mL/kg of body weight weekly for 8 weeks); the Dox (D) group received an intraperitoneal injection of Dox (1 mg/kg of body weight weekly for 8 weeks); the Dox + peptide G (D + G) group received an intraperitoneal injection of Dox (1 mg/kg of body weight weekly for 8 weeks) and a subcutaneous injection of peptide G (50 nmol/kg of body weight daily for 8 weeks); and peptide G (G) group, a subcutaneous administration of G (50 nmol/kg of body weight daily for 8 weeks). Before the study (initial state) and after the 8-week experiment, the animals were weighed; the activity of CK-MB and the level of thiobarbituric acid reactive substances (TBARS) in blood plasma were determined; and the cardiac function was evaluated by echocardiography. At the end of the 8-week experiment, the beating hearts of the animals anesthetized with urethane (120 mg/kg) in each group were excised to subsequently determine the levels of myocardial metabolites and TBARS, as well as perform a morphological examination.

**Modified N-terminal galanin fragment G**

Peptide G, the modified galanin fragment (2–15) (H-Trp-Thr-Leu-Asn-Ser-Ala-Gly-Tyr-Leu-Leu- Gly-Pro-βAla-His-OH, Mw ~ 1499.7 g/mol), was synthesized by Fmoc (9-fluoroenylmethyloxycarbonyl) solid-phase peptide synthesis using a Tribute-UV peptide synthesizer (Protein Technologies Inc., USA) in the stepwise automatic mode [7]. Peptide G was purified by reversed-phase high-performance liquid chromatography. Its structure was confirmed by both 1H NMR (WM-500 Bruker spectrometer, Germany) and mass spectrometry (MALDI-TOF, Bruker Daltonics, Germany).

**Transthoracic echocardiography**

Echocardiography was performed on a Vevo 1100 Visual Sonic high-frequency ultrasound system (FUJILM, the Netherlands) equipped with a linear sensor of 13–24 MHz and a maximum image depth of 40 mm. The study was performed on anesthetized rats (Zoletil 100, Virbac Sante Animale, France, 5 mg/kg) via parasternal access along the short and long axes. The diastolic and systolic dimensions of the LV were measured in the B-mode; the resulting values were used to calculate the LV parameters in diastole and systole, as well as the ejection fraction (EF) and shortening fraction (SF).

**Determination of the metabolite content in cardiac tissue**

In each group, a portion of the cardiac tissue frozen in liquid nitrogen was quickly homogenized in cooled 6% HClO_4_ (10 mL/g of the tissue) using an Ultra-Turrax T-25 homogenizer (IKA-Labortechnik, Staufen, Germany). The homogenates were centrifuged at 2800*g* for 10 min at 4°C. The supernatants were neutralized with 5M K_2_CO_3_ to pH 7.4, and the extracts were centrifuged after cooling to remove the KClO4 precipitate. Protein- free extracts were stored at -20°C until the determination of the metabolite contents. The dry weights of the tissue were determined by weighing a portion of the pellets after extraction with 6% HClO_4_ and drying overnight at 110°C. The levels of adenine nucleotides (ATP, ADP, and AMP), phosphocreatine (PCr), creatine (Cr), glucose, lactate, glutamic acid (Glu), aspartic acid (Asp), alanine (Ala), and ammonia in the extracts were determined enzymatically [10] using a Shimadzu UV-1800 spectrophotometer (Japan).

**Determination of the activity of antioxidant enzymes and TBARS in the heart**

The remaining rat hearts frozen in liquid nitrogen were homogenized in a 50 mM Na-phosphate buffer, pH 7.4, (10 mL/g of tissue) using an Ultra-Turrax T-25 homogenizer (IKA-Labortechnik, Germany) and centrifuged in a Sigma 3-16 KL centrifuge (USA) at 1000*g *and 4°C for 10 min. The content of TBARS was determined in homogenates. The activity of Cu,Zn-superoxide dismutase (Cu,Zn-SOD), catalase (CAT), and glutathione peroxidase (GSH-Px) was measured in supernatants . Protein in supernatants was determined by the method of Lowry. All measurements were performed on a Shimadzu 2600 spectrophotometer (Japan). The TBARS content was determined in the reaction with 2-thiobarburic acid (2TBA) at λ = 532 nm [11]. The activity of Cu,Zn-SOD was determined according to the suppression of the reduction rate of nitro blue tetrazolium during superoxide anion radical generation in the oxidation of xanthine with xanthine oxidase at λ = 560 nm [12]. The activity of CAT was measured according to the consumption rate of hydrogen peroxide (H_2_O_2_) at 20°C for 1 min, taking the molar extinction coefficient of H_2_O_2_ as 43.6 M-1cm-1 [13]. The activity of GSH-Px was determined according to the oxidation rate of NADPH in the conjugated glutathione reductase system at λ = 340 nm. H_2_O_2_ was used as a substrate. The reaction was carried out in the presence of 3 mM sodium azide to inhibit CAT [14].

**Assessment of damage to cell membrane and plasma concentration in TBARS**

The activity of CK-MB was determined on a Shimadzu UV-1800 spectrophotometer (Japan) at λ = 340 nm using BioSystems kits (Spain). The plasma concentration in TBARS was determined according to the formation of a colored complex in the reaction with 2TBA, which was extracted from the reaction mixture with butanol [15].

**Histopathological evaluation**

The hearts of the animals in each group were fixed in 10% buffered formalin (pH 7.4) for 24 h and processed for histopathological examination. A 2-mm-thick slice perpendicular to the longitudinal organ and containing the free LV wall was excised from the upper part of LV. The samples were dehydrated by passage through increasing concentrations of alcohol (70%–100%), cleared by passage through xylol, and finally embedded in paraffin blocks. 5-μm-thick paraffin sections were stained with haematoxylin and eosin (HE) to demonstrate the general histological structure and with haematoxylin basic fuchsine picric acid (HBFP) to detect fuchsinophilic ischemic cardiomyocytes [16]. Microscopic examination of the histological specimens was performed on a DM2500 Leica light microscope (Germany).

**Statistical analysis**

All statistical analyses were performed using Sigma- Plot version 12 (Systat Software Inc, San Jose, CA). The data are presented as a mean ± SEM. The results were analyzed by one-way ANOVA, followed by Bonferroni multiple range test post-hoc analysis to calculate the differences between more than two groups. Comparisons between two groups involved the use of a Student’s unpaired t-test. *P* < 0.05 was regarded as significant.

## RESULTS

**Effect of peptide G on cardiotoxicity and oxidative stress**

**Fig. 1:**
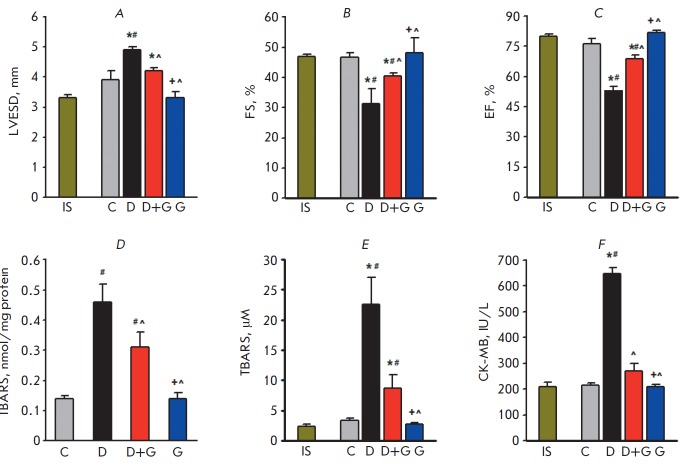
Echocardiography variables and indices of oxidative stress and cell membrane damage in the studied groups.* A *– Left ventricular end-systolic diameter (LVESD). *B *– Fractional shortening (FS) was calculated as (LVEDD – LVESD)/ LVEDD × 100%, where LVEDD is the left ventricular end-diastolic diameter. *C *– Ejection fraction (EF) was calculated as (LVEDV – LVESV)/LVEDV × 100%, where LVEDV is the left ventricular diastolic volume and LVESV is the left ventricular systolic volume. *D *– Myocardial content of thiobarbituric acid reactive substances (TBARS). *E *– TBARS concentration in blood plasma. *F *– The plasma activity of creatine kinase-MB (CK-MB). IS – initial state; C – control; D – doxorubicin; D + G – doxorubicin + peptide G; G – peptide G. Values are expressed as the mean ± SEM for groups consisting of 12 animals each. *P* < 0.05 vs. ^*^IS, ^#^C, ^^^D, ^+^D ^+^G

The echocardiographic study of control group animals revealed no changes in the heart rate and LV contractility indices compared to the initial state after 8 weeks of observation. By the end of the eighth week, the Dox-treated group exhibited a pronounced cardiomyopathy (CM) as evidenced by a significant increase in the left ventricular end-systolic diameter (LVESD) and a reduction in fraction shortening (FS) and ejection fraction (EF) to 67 and 69% of the baseline level, respectively (*Fig. 1 A, B, C*). Coadministration of peptide G and Dox significantly reduced LVESD and increased SF and EF compared with these parameters in group D. The development of CM induced by Dox was accompanied by the activation of LP and damage to the cell membrane. After 8 weeks into the study, the TBARS content in myocardial tissue and the blood plasma of Dox-treated rats was higher than that in the control animals (*Fig. 1 D, E*). Coadministration of peptide G and Dox significantly reduced these indices. At the end of the study, CK-MB activity in group D was almost three times higher than that in the control group (*Fig. 1F*). Administration of peptide G simultaneously with Dox reduced the activity of plasma CK-MB almost to the value in the control group. Treatment of the animals with peptide G for 8 weeks did not affect echocardiographic variables, plasma, or myocardial TBARS contents, or the plasma CK-MB activity as compared with the initial values. Thus, peptide G attenuated the damaging effect of Dox on the heart by reducing LV dysfunction, oxidative stress, and damage to sarcolemma.

**The effect of peptide G on the morphological changes in cardiac tissue**

**Fig. 2:**
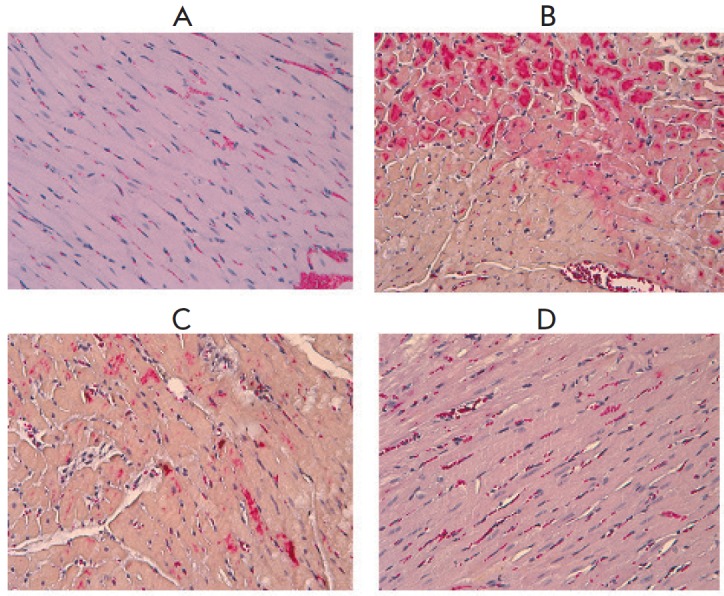
The effects of peptide G on histological changes in the hearts of rats treated with Dox. *A *– Control, HE, ×200. Slight plethora and erythrocyte stasis. *B *– Dox group, HBFP, ×200. Extensive fuchsinophilia of cardiomyocytes.* C *– D + G group, HBFP, ×200. Groups of single cardiomyocytes with cytoplasmic fuchsinophilia.* D *– G group, HBFP, ×200. Slight erythrocyte stasis, lack of fuchsinophilic cardiomyocytes

In the control group, mild plethora and moderate erythrocyte stasis in capillaries were observed after HE staining (*Fig. 2A*). No fuchsinophilia foci were detected after staining with HBFP, thus suggesting the absence of myocardial damage and contracture [17]. Most of the animals in group D showed a marked erythrocyte stasis in the capillaries and plethora. When stained with HBFP, multiple areas of diffuse and focal cardiomyocyte fuchsinophilia were found in half of the animals treated with Dox (*Fig. 2B*). In the D+G group, single cardiomyocytes with fuchsinophilia were observed only in one case with a marked erythrocyte stasis and plethora (*Fig. 2C*). No fuchsinophilia foci were identified in most of the hearts in this group. Like in the control group, slight erythrocyte stasis was observed after administration of peptide G alone. In most of the hearts of group G animals, no fuchsinophilia foci were identified (*Fig. 2D*). Single fuchsinophilic cardiomyocytes were detected only in one animal by HBFP staining. Thus, administration of peptide G reduced the degree of histopathological changes in the heart of rats treated with Dox.

**Effect of peptide G on the activity of myocardial antioxidant enzymes**

Administration of Dox reduced the activity of GSH-Px and led to a clear tendency towards a decreased activity of Cu, Zn-SOD and increased activity of CAT compared to the control group (*Table 1*). Coadministration of Dox and peptide G significantly increased the activities of Cu,Zn-SOD and GSH-Px and slightly enhanced the activity of CAT (*P *= 0.082) compared with these indices in group D. Treatment with peptide G for 8 weeks did not affect the activity of antioxidant enzymes compared to the control group.

**Table 1:** Effects of doxorubicin and peptide G on the activity of antioxidant enzymes in the rat heart after 8 weeks of study

Group	Cu,Zn-SOD	CAT	GSH-Px
C	220.75 ± 17.92	16.73 ± 0.43	0.21 ± 0.01
D	165.50 ± 22.77	21.50 ± 0.50^*^	0.16 ± 0.01^*^
D + G	259.64 ± 13.78^#^	23.67 ± 1.49^*^	0.20 ± 0.01^#^
G	227.55 ± 19.31	19.13 ± 1.08	0.19 ± 0.01

Values are expressed as the mean ± SEM for groups consisting of 6 animals each and expressed in IU/mg protein. C – control; D – doxorubicin; D + G – doxorubicin + peptide G; G – peptide G. *P* < 0.05 vs. * C; # D.

**Effect of peptide G on myocardial energy metabolism**

After an 8-week study of group D rats, the myocardial adenine nucleotide pool (ΣAN) and adenylate energy charge (AEC) of cardiomyocytes were significantly lower compared to the control due to a reduction in the ATP content (*Table 2*). In addition, the myocardial PCr content in the Dox-treated group was down by 50%. This was the reason for the significant decrease in total creatine (ΣCr), since the Cr level was not affected by Dox. Administration of peptide G, together with Dox, increased the levels of ATP and ADP, resulting in a 1.5- fold increase in ΣAN to a value that did not differ from the control value. In animals of the D+G group, the myocardial contents of PCr and ΣCr were higher than those in the D group and did not differ significantly from the control values. Since intracellular ΣCr losses are attributed to damage to sarcolemma [18], a higher ΣCr content in the D + G group is an indication that administration of protein G is associated with a lower degree of cell membrane injury.

**Table 2:** Effects of doxorubicin and peptide G on myocardial energy state after 8 weeks of study

	C	D	D+G	G
ATP	18.84 ± 1.17	11.83 ± 1.33^*^	16.31 ± 1.32^#^	18.91 ± 1.97^#^
ADP	5.47 ± 0.11	5.60 ± 0.35	7.28 ± 0.47^*#^	6.30 ± 0.33^*^
AMP	0.92 ± 0.06	1.14 ± 0.16	1.58 ± 0.15^*^	1.50 ± 0.10^*^
ΣAN	25.24 ± 1.22	18.56 ± 1.66^*^	25.18 ± 1.50^#^	25.71 ± 1.13^#^
AEC	0.86 ± 0.01	0.78 ± 0.02^*^	0.79 ± 0.01^*^	0.85 ± 0.02
PCr	22.57 ± 1.52	12.08 ± 1.25^*^	17.74 ± 1.14^*#^	20.66 ± 2.04^#^
Cr	34.94 ± 2.64	38.34 ± 3.80	38.24 ± 3.89	37.43 ± 2.67
ΣCr	57.51 ± 1.67	50.42 ± 2.26^*^	55.98 ± 2.12	58.09 ± 2.81^#^

Values are expressed as a mean ± SEM for groups consisting of six animals each and expressed in μmol/g dry weight for metabolites. ΣAN = ATP + ADP + AMP; AEC = (ATP + 0.5ADP)/ΣAN; ΣCr = PCr + Cr. C – control; D – doxorubicin; D + G – doxorubicin + peptide G; G – peptide G. *P* < 0.05 vs. *C; #D.

**Effect of peptide G on the myocardial content of carbohydrate and nitrogenous metabolites**

**Fig. 3:**
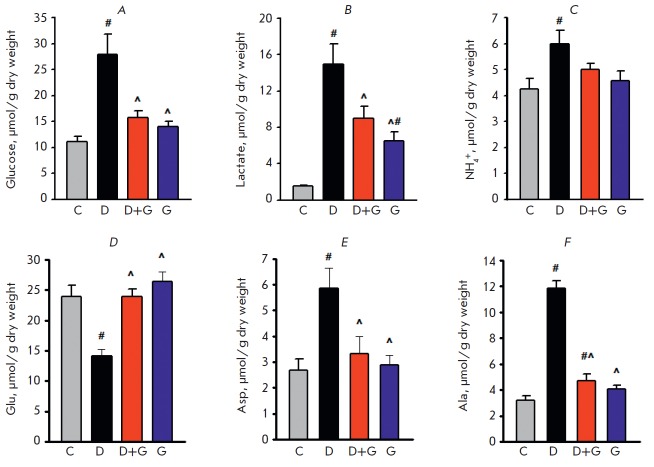
Myocardial levels of glucose, lactate, glutamic acid (Glu), aspartic acid (Asp), alanine (Ala), and ammonia (NH4 +) in the studied groups after 8 weeks of experiment. C – control; D – doxorubicin; D + G – doxorubicin + peptide G; G – peptide G. Values are expressed as the mean ± SEM for groups consisting of 6 animals each. *P* < 0.05 vs. # C, ^D

A decrease in β-oxidation of fatty acids and a concomitant increase in myocardial glucose uptake induced by treatment with Dox [19] enhanced anaerobic glycolysis and raised the glucose and lactate levels in the hearts of group D rats after the 8 weeks of experiment compared to the control group (*Fig. 3 A, B*). Coadministration of peptide G and Dox reduced the glucose level to a value close to the control value, while simultaneously lowering lactate accumulation compared with that in group D. When only peptide G was administered, the glucose level in the heart did not significantly differ from that in the control group, while the lactate level remained higher than that in the control group.

We studied the effects of Dox and peptide G on the changes in the metabolism of key myocardial amino acids: glutamic acid (Glu), aspartic acid (Asp), alanine (Ala), and ammonia. The disruptions in the aerobic energy supply to the heart caused by exposure to Dox increased the rates of Glu catabolism and Ala formation. After the 8 weeks, the Glu content in group D was significantly reduced, while the Ala content was increased compared with these indicators in the control group due to transamination of glycolytic pyruvate. As a result of coupled transamination of Glu and oxaloacetate, the Asp content in group D had doubled compared with the control group (*Fig. 3 D, E, F*). Administration of peptide G in the D + G group restored the Glu content, while the Ala content fell to values not differing from those in the control group. Meanwhile, the Asp level in the D + G group decreased to the control value. Administration of Dox to the rats significantly increased the myocardial content of ammonia compared with the control by the end of the study (*Fig. 3C*). Administration of peptide G reduced the ammonia content to a value not different from that of the control. Thus, the improved energy state of a heart damaged by Dox due to the administration of peptide G prevented changes in the metabolism of Glu, Asp, and Ala and reduced ammonia accumulation in the myocardium. Administration of peptide G for 8 weeks did not lead to changes in the levels of these nitrogenous compounds compared with the control values.

## DISCUSSION

Our findings demonstrate the protective effect of peptide G in a rat model of CM induced by chronic Dox administration. Reduction in LV dysfunction and LV remodeling with coadministration of Dox and peptide G was accompanied by significant improvement in the energy supply to cardiomyocytes. This was evidenced by the higher levels of ATP, ΣAN, and PCr in the heart. accompanied by a reduced accumulation of glucose and lactate. It is noteworthy that Dox-induced CM increased the rate of myocardial Glu catabolism, which was accompanied by Ala and Asp formation and accumulation of cytotoxic ammonia. Such changes in the metabolism of these amino acids are typically found when oxidative phosphorylation cannot provide the required formation of ATP that leads to the activation of glycolysis and utilization of high-energy phosphate reserves [20, 21]. The coupled transamination of Glu and Asp and the formation of Ala mobilize substrate phosphorylation in mitochondria at the succinate level, thus compensating for the inhibition of oxidative phosphorylation. Moreover, the loss of the intracellular Glu pool (which is involved in support to the ATP level) and the production of Ala are closely related to the myocardial energy state. The improvement in aerobic metabolism under the action of peptide G in a heart damaged by Dox restored the normal myocardial levels of Glu, Asp, and Ala and reduced ammonia formation. Such shifts in intracellular myocardial metabolism are indicative of a reduction in the NADH/NAD^+^ ratio in the cytosol, a normalization of the function of the malate-aspartate shuttle and the tricarboxylic acid cycle [22, 23]. They are consistent with higher respiratory control in cardiac mitochondria on NAD^+^-dependent substrates (Glu and malate), which were found after coadministration of Dox and peptide G to rats [9].

Control over the ammonia content in the cardiac muscle characterized by intense aerobic metabolism is of particular importance. This is related to its ability to (i) inhibit the decarboxylation of α-ketoacids in the Krebs cycle and protein synthesis; (ii) shift the direction of the glutamate dehydrogenase reaction towards Glu formation, thus inhibiting the catabolism of amino acids; and (iii) disturb the active transfer of monovalent cations and change the intracellular pH [24, 25]. The decrease in the intracellular level of ammonia, which has a toxic effect on oxidative metabolism, is caused by the improvement in the myocardial metabolism that is due to the administration of peptide G. This effect is most likely to result from the activation of ammonia binding in ATP-dependent reactions of glutamine and asparagine formation, as well as the reduced adenine nucleotide degradation [26, 27]. Thus, peptide G corrected the myocardial energy, as well as the carbohydrate and nitrogenous metabolism during CM.

**Fig. 4:**
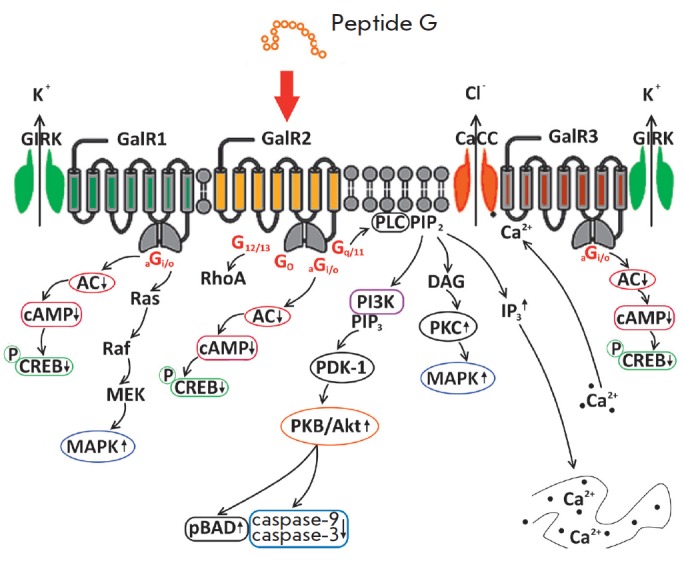
Intracellular signaling pathways activated by peptide G, a pharmacological galanin receptor agonist (adapted from Runesson [31]). AC – adenylate cyclase; (p)BAD – (phosphorylated) BCl-2 associated death promoter; CaCC – Ca^2+^-activated chloride channel; cAMP – 3’,5’-cyclic adenosine monophosphate; (p)CREB – (phosphorylated) cAMP response element binding protein; DAG – diacylglycerol; GIRK – G protein-coupled inwardly rectifying potassium channel; IP3 – inositol triphosphate; MAPK – mitogen- activated protein kinase; MEK – mitogen extracellular kinase; PDK-1 – phosphoinositide-dependent protein- kinase I; PIP2 –phosphatidylinositol biphosphate; PIP3 – phosphatidylinositol triphosphate; PI3K – phosphatidylinositol 3-kinase; PKB – protein kinase B; PLC – phospholipase C; RhoA – Ras homolog gene family, member A

At the moment, all three subtypes of galanin receptors (GalR1, GalR2 and GalR3) that also exist in the heart have been cloned and pharmacologically characterized. The N-terminal fragment of the peptide is responsible for its binding to receptors; the first 15 amino acid residues of this fragment are conserved in most animal species and humans [28]. The effect of peptide G on the metabolic state of a damaged heart is probably related to the fact that it binds mainly to the GalR2 receptor, coupled with various types of G proteins; this binding activates the mechanisms of cell protection (*Fig. 4*). Activation of all subtypes of galanin receptors through Gi/o proteins reduces cAMP and inhibits the phosphorylation of the CREB transcription factor (the cAMP-response element binding protein). This, in turn, increases the expression of the GLUT4 transporter and its translocation to the sarcolemma, thus stimulating Cardiomyocyte glucose uptake and oxidation by cardiomyocytes. Triggering of this mechanism is of critical importance in the reduction in ATP formation [29]. Coupling of the GalR2 receptor with the Gq/11 protein activates phospholipase C and regulates Ca^2+^ homeostasis through hydrolysis of phosphatidylinositol diphosphate, which improves the inotropic properties of the heart [30]. The downstream pathways of this signaling cause phosphorylation of protein kinase B (Akt) leading to the inhibition of pro-apoptotic BAD/BAX proteins and activation of caspase-3 and caspase-9 [31]. The reduced cardiomyocyte apoptosis, including that induced by Dox administration, is generally combined with a reduction in the degree of irreversible myocardial injury and an enhancement of cardiac contractile function in* in vivo *models [32]. Activation of the GalR1 and GalR2 receptors stimulates the signaling pathways activated by mitogen-activated protein kinases (MEK1/2 and ERK1/2), thus leading to the inhibition of mitochondrial permeability transition pore (mPTP) opening. This mechanism is responsible for cell survival and mobility [33]. Furthermore, the upregulation of ERK phosphorylation promotes an increased expression of peroxisome proliferator-activated receptors (PPARs), the transcription factors that control energy metabolism, including the PPARγ expression that stimulates glucose uptake and oxidation by cardiomyocytes [34]. Hence, it follows that peptide G is capable of triggering various mechanisms that contribute to the protective cardiometabolic effects in CM.

**Fig. 5:**
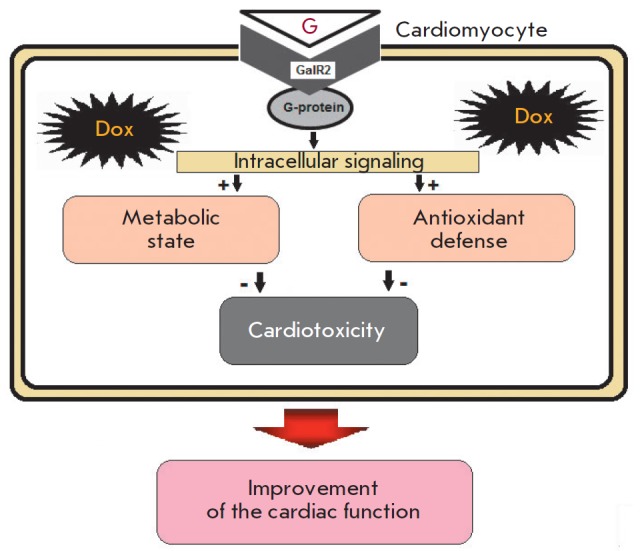
Galanin GalR2 receptor activation by peptide G reduces myocardial systolic dysfunction in Dox-induced CM thanks to an improved metabolic and antioxidant state of the heart

Oxidative stress is one of the leading factors of Dox-induced cardiotoxicity [2, 4]. In our study, this was confirmed by the increase in TBARS content at both the systemic and organ levels, as well as the increased plasma activity of CK-MB, a specific necrosis marker, in animals treated with Dox. In order to understand the features of oxidative stress in the heart under the influence of Dox, we studied the activities of the key enzymes of the myocardial antioxidant defense system: Cu,Zn-SOD, CAT, and GSH-Px. In Dox-treated rats, activity was significantly reduced only for GSH-Px, while that of Cu,Zn-SOD decreased slightly and the activity of CAT increased compared to the control. Such different responses of antioxidant enzymes are associated with the fact that we used the low cumulative dose of Dox (8 mg/kg) in our experiments. Dox, generating a small amount of ROS, may not affect the activity of antioxidant enzymes and even activate a number of signaling pathways, thus inducing antioxidant protection. Similar data were obtained for Dox-induced oxidative stress models in animals of various species [35, 36]. It is important that administration of peptide G to animals in which the heart had been damaged by Dox significantly reduced the content of LP products in the heart and plasma and improved cardiomyocyte membrane integrity. This reduction in oxidative stress was accompanied by increasing activity of Cu,Zn-SOD and GSH-Px, which indicates an increase in antioxidant protection. It should be noted that intracellular Cu,Zn-SOD controls the formation not only of ROS, but highly reactive peroxynitrite as well, thus limiting nitrosyl stress [37]. The observed upregulation of Cu,Zn-SOD, CAT, and GSH-Px might be related to the increased gene expression of these enzymes under the influence of peptide G. Some peptides are known to possess radical scavenging properties and to be able to inhibit LP [38]. However, no data on the direct antioxidant action of galanin peptides or peptides containing a carnosine sequence at their C-terminal end are available in the literature. There is no doubt that the galanin- induced mechanisms of ischemic heart adaptation to oxidative stress need further study. We believe that improvement in energy supply to cardiomyocytes and enhancement of the enzymatic antioxidant defense are the main factors responsible for the pharmacological effectiveness of peptide G and the decrease in cardiotoxicity caused by prolonged administration of Dox. A diagram illustrating the cardioprotective effect of this synthetic galanin receptor agonist is shown in *Fig. 5*.

We previously studied the toxicity of peptide G in BALB/c mice. The administration of peptide G caused no signs of intoxication or death of animals during the 14 days of observation [39]. It is noteworthy that the highest tested dose of peptide G (520 mg/kg) was many times higher than the cumulative dose used in this study with Dox (4.2 mg/kg). These data are indicative of the good tolerance of peptide G and the potential for preclinical studies of this drug, which has a broad range of protective effects in Dox-induced CM.

## CONCLUSIONS

Although next-generation anthracyclines with reduced cardiotoxicity (epirubicin and idarubicin) have been developed, Dox remains a drug with a high antitumor effect. In turn, this makes it necessary to reduce the damage to the cardiovascular system caused during its administration as a chemotherapy agent. Our findings clearly indicate that CM induced by chronic administration of Dox in rats can be corrected using peptide G, a pharmacological galanin receptor agonist. The attenuation of Dox-induced cardiotoxicity with peptide G was confirmed by the decreased LV systolic dysfunction and reduced plasma activity of CK-MB, a specific marker of myocardial damage. These beneficial effects are directly associated with the reduction in oxidative stress and improvement in the metabolic and antioxidant state of the heart. To further understand the mechanisms of action of peptide G, the role of the activation of galanin receptors by this ligand and the signal transduction pathways in the cardiomyocytes need further study. These data provide an incentive to explore the feasibility of using pharmacological ligands of galanin receptors in oncology in order to reduce the toxicity of anthracycline antibiotics. They also indicate the usefulness of preclinical studies of peptide G, which can be used as a potential anti-ischemic, membrane- stabilizing, and antioxidant drug.

## Abbreviations

2TBA: 2-thiobarbituric acid
AEC: adenylate energy charge
Ala: alanine
Asp: aspartic acid
ROS: reactive oxygen species
Dox: doxorubicin
I/R: ischemia/reperfusion
CK-MB: creatine kinase-MB
CM: cardiomyopathy
Cr: creatine
LV: left ventricle
LP: lipid peroxidation
TBARS: thiobarbituric acid reactive substances
EF: ejection fraction
PCr: phosphocreatine
FS: fractional shortening
EchoCG: echocardiography
CAT: catalase
Cu,Zn-SOD: Cu,Zn superoxide dismutase
GSH-Px: glutathione peroxidase
PPARs: peroxisome proliferator-activated receptors
ΣAN: adenine nucleotide pool
ΣCr: total creatine
